# Evaluating a Targeted Minimum Loss-Based Estimator for Capture-Recapture Analysis: An Application to HIV Surveillance in San Francisco, California

**DOI:** 10.1093/aje/kwad231

**Published:** 2023-11-17

**Authors:** Paul Wesson, Manjari Das, Mia Chen, Ling Hsu, Willi McFarland, Edward Kennedy, Nicholas P Jewell

**Keywords:** bias, capture-recapture method, hidden populations, human immunodeficiency virus, machine learning, prevalence estimation, SuperLearner, targeted minimum loss-based estimation

## Abstract

The capture-recapture method is a common tool used in epidemiology to estimate the size of “hidden” populations and correct the underascertainment of cases, based on incomplete and overlapping lists of the target population. Log-linear models are often used to estimate the population size yet may produce implausible and unreliable estimates due to model misspecification and small cell sizes. A novel targeted minimum loss-based estimation (TMLE) model developed for capture-recapture makes several notable improvements to conventional modeling: “targeting” the parameter of interest, flexibly fitting the data to alternative functional forms, and limiting bias from small cell sizes. Using simulations and empirical data from the San Francisco, California, Department of Public Health’s human immunodeficiency virus (HIV) surveillance registry, we evaluated the performance of the TMLE model and compared results with those of other common models. Based on 2,584 people observed on 3 lists reportable to the surveillance registry, the TMLE model estimated the number of San Francisco residents living with HIV as of December 31, 2019, to be 13,523 (95% confidence interval: 12,222, 14,824). This estimate, compared with a “ground truth” of 12,507, was the most accurate and precise of all models examined. The TMLE model is a significant advancement in capture-recapture studies, leveraging modern statistical methods to improve estimation of the sizes of hidden populations.

## Abbreviations

CI: confidence interval
HIV: human immunodeficiency virus
SFDPH: San Francisco Department of Public Health
TMLE: targeted minimum loss-based estimation

Establishing the true denominator of a population at risk is critical for determining rates of disease burden and acquisition, allocating resources, and setting appropriate public health priorities and policies. Multiple systems estimation, often referred to as capture-(mark-)recapture, is commonly used in epidemiology to estimate the denominator for the population at risk and to correct the underascertainment of cases in disease surveillance (1, 2). The capture-recapture method estimates an unobserved population size based on the number of people already observed on multiple overlapping and incomplete lists of the target population (3–5). The greater the lists’ overlap, the smaller the unobserved population; conversely, the smaller the overlap, the greater the unobserved population. In public health applications, lists can be administrative records from medical centers, disease registries, and surveillance systems, or direct samples of the target population from cross-sectional surveys, among other possibilities (6). A key assumption for valid estimation is that the lists are statistically independent of one another. Presence on one list cannot increase (positive dependence) or decrease (negative dependence) the probability of presence on another list. Positive dependence will underestimate the true population size, while negative dependence will result in overestimation. There are 3 other formal assumptions: the population is “closed” (there are no entries or exits between capture occasions); there is accurate matching of individuals on multiple lists/captures; and, for each list, everyone has the same probability of capture (capture homogeneity) (3). An additional, implicit assumption is random assignment to lists, meaning the population observed on the lists is reflective of the unobserved population.

The popularity of the capture-recapture method persists in epidemiology despite a fundamental limitation: The population size estimate parameter is not identified without untestable assumptions (7, 8). In capture-recapture modeling, there is always 1 fewer degree of freedom available than is needed to estimate all parameters in the model. Variations in modeling always impose a constraint in order to identify the unobserved population size. In log-linear regression, a common model form for capture-recapture due to the data often being represented as cross-classified categorical data (9), the constraint is often that *k*-way interaction is 0 (where *k* is the number of lists). This is a strong assumption when using only 2 lists, as it assumes list independence, but it may become more reasonable with additional lists. Additional lists confer greater statistical flexibility because more degrees of freedom allow modeling of lower-level list-dependence through interaction terms.

Despite their broad implementation, log-linear models are also criticized for producing implausible or unreliable estimates (10–12). Using at least 3 lists, multiple log-linear models may be fitted, each accounting for a different combination of potential list dependencies. Model selection is often achieved by selecting the model with the lowest information criterion. This approach presents a single model estimate, ignoring potentially plausible estimates from other models with an approximately equally good fit to the data. The information criterion does not always uniquely identify the best model; two models can have the same information criterion and give very different population size estimates (12). Furthermore, model selection based on the information criterion can be extremely sensitive to cell sparsity (i.e., few people observed in cells corresponding to different capture patterns). Because coefficients in the model are jointly determined, searching the extreme regions of the parameter space to maximize the likelihood of probabilities for the sparse cells also impacts predictions for other cells (11, 13). This sometimes results in implausibly large size estimates and a deceptively small information criterion, making it the (statistically) preferred model.

The log-linear modeling approach is arguably statistically inefficient because degrees of freedom are spent estimating parameters already observed and not of interest (i.e., much of the probability distribution). The observed data, the number “captured” on each combination of lists, are fitted to a log-linear model to project to the intercept and estimate the number not seen on any list. Forcing the data to fit the log-linear functional form is another potential limitation. This model is chosen out of convenience but may not reflect the true statistical model. Model misspecification can yield biased estimates. These limitations to conventional log-linear modeling motivated a targeted minimum loss-based estimation (TMLE) approach to capture-recapture, paired with semiparametric modeling using machine learning techniques.

In this paper, we describe and evaluate a novel TMLE capture-recapture estimator using data from the San Francisco Department of Public Health (SFDPH) human immunodeficiency virus (HIV) surveillance registry. We applied the TMLE estimator to data collected from medical center lists reporting cases to the SFDPH to estimate the total number of people living with HIV in San Francisco and accessing HIV-related medical care in 2019. We compared TMLE population size estimates with estimates from conventional capture-recapture models and the likely population size (ground truth), based on SFDPH surveillance data.

## METHODS

### Study population

Our target population was the number of San Francisco residents living with HIV as of December 31, 2019. This population included out-of-jurisdiction residents at the time of HIV diagnosis who later moved to and received care in San Francisco by the end of 2019. Excluded from this population were San Francisco residents at the time of diagnosis who then moved away from San Francisco by the end of 2019. Deidentified data were pulled from the San Francisco HIV case registry data and data from the San Francisco HIV laboratory data management system as of January 7, 2022. The data extraction included 3 indicator variables for whether a patient was observed on any of 3 chosen lists feeding into the surveillance system: the ward 86 HIV clinic at Zuckerberg San Francisco General Hospital (the largest public HIV care provider in the city), the SFDPH Laboratory (the public health laboratory covering patients for whom HIV-related laboratory tests are ordered), and the Tom Waddell Urban Health Clinic (a community clinic serving a diverse patient population, including racial minorities, transgender people, and people who are marginally housed). These lists were chosen for their relative size and the diverse patient populations they collectively contribute to SFDPH surveillance. For the capture-recapture analysis, we only considered patients who appeared on at least 1 of the 3 lists, using the full data set from the HIV case registry as the “ground truth” for comparison to assess the performance of various estimators.

Additionally, we extracted the following covariates believed to influence a person’s probability of selection on one or multiple lists: race and ethnicity, birth sex, age in 2019, age at the time of HIV diagnosis, HIV transmission risk category, indicator of new diagnosis in 2019, and viral suppression status.

### TMLE overview and application to capture-recapture

In contrast to the classic log-linear model discussed above, Das et al.’s TMLE approach (14) reformulates the target parameter, Ψ, as the probability of being observed on any list (i.e., the capture probability). Dividing the observed *n* by Ψ yields the estimated population size. Das et al.’s approach, like other approaches (15), requires a single identifiability assumption; with a pair of lists, selection on one list is conditionally independent of selection on another list, given a set of covariates, akin to classical confounding. Das et al. estimate Ψ by

$$ \hat{\psi} {\mkern6mu} ={\left[{\mathbb{Q}}_N\left\{\frac{1}{\hat{\gamma} (X)}\right\}\right]}^{-1}, $$

where ${\mathbb{Q}}_N$ is the empirical measure of the observed (biased) data distribution $\mathbb{Q}$ of capture patterns. $\hat{\gamma}$ depends on conditional capture probabilities, given a level of *X*, as follows:

$$ \hat{\gamma} {\mkern6mu} (X)=\frac{{\hat{q}}_{12}(X)}{{\hat{q}}_1(X){\hat{q}}_2(X)}, $$

where ${\hat{q}}_1$, ${\hat{q}}_2$, and ${\hat{q}}_{12}$ are observational probabilities (*q*-probabilities) for being captured by list 1, by list 2, and by both lists 1 and 2, respectively. **X** is a vector of covariates that may influence these *q*-probabilities.

TMLE is a doubly robust, maximum-likelihood–based estimation method that optimizes the bias-variance tradeoff through a “targeting” mechanism (16) that has been widely used to address causal inference problems in epidemiology. TMLE has been described in detail elsewhere (17). Briefly, the TMLE framework in this context begins with an initial estimation of the *q*-probabilities *q*_1_(*X*), *q*_2_(*X*), and *q*_12_(*X*) (i.e., *P*(*Y*_1_ = 1|*X*), *P*(*Y*_2_ = 1|*X*), and *P*(*Y*_1_*Y*_2_ = 1|*X*), respectively). In the “targeting” step for TMLE, the nuisance parameters are estimated as part of the “clever covariates,” the coefficients of which are used to update the initial estimates of *q*_1_(*X*), *q*_2_(*X*), and *q*_12_(*X*). This process continues iteratively according to a stopping rule and reduces bias in the initial estimate of Ψ = *P*(*Y* ≠ 0). The final updated estimates of *q*_1_(*X*), *q*_2_(*X*), and *q*_12_(*X*) are then used to calculate the target parameter Ψ = *P*(*Y* ≠ 0). These steps are outlined in Appendix 1. TMLE benefits from statistical flexibility by incorporating various algorithms and machine learning methods (e.g., cross-validation) to model complex relationships in the data without making overly restrictive assumptions about functional form.

As a corollary to the doubly robust properties of TMLE, for the TMLE capture-recapture model, if either estimate of *q*_1_ or *q*_2_ has small error and if either estimate of *q*_12_ or γ has small error, then the overall error for ${\hat{\psi}}_{\mathrm{tmle}}$ (the TMLE for $ \hat{\psi}$) will be just as small, even if the other estimates have large errors or are misspecified (14).

If more than 2 lists are available, ${\hat{\psi}}_{\mathrm{tmle}}$ can be estimated for all pairwise combinations of lists. The lists not used to directly estimate the *q*-probabilities are treated either as additional lists or as covariates. If the former, the additional list(s) provide information to refine *q*-probabilities; the model now becomes aware of additional people who are part of the target population yet may not be observed on either of the 2 primary lists, updating the capture probabilities for those two lists. If the latter, the additional list(s) may be used alongside other covariates in the vector **X** to make the 2 primary lists conditionally independent from one another.

The TMLE model for capture-recapture draws upon multiple algorithms to model the relationship between conditioning covariates and estimate the *q*-probabilities. Currently, these algorithms include logistic regression, generalized additive models, Ranger (a random forest algorithm), multinomial logistic regression, and rangerlogit (an ensemble model using Ranger and logistic regression). SuperLearner, an ensemble machine learning method, is also a feature of the model, permitting multiple algorithms to be used at once to flexibly model these relationships and parameters (18). Cross-validation is used to prevent overfitting and flexibly model the data. Ninety-five percent confidence intervals (CIs) are estimated on the basis of the efficient influence function.

Additionally, the sizes of population subgroups may be estimated on the basis of categorical variables included in **X**.

### TMLE capture-recapture model simulations

Initial estimation of population sizes using TMLE revealed sensitivity to a so-called margin setting. The margin setting is a function that prevents searching the extremes of the parameter space to estimate small *q*-probabilities. This will likely occur with small cell sizes.

We conducted simulations to inform the optimal margin setting. All simulations included 3 lists with different marginal capture probabilities for each list. To match the SFDPH data, the true population size was set at 12,500 for all simulations.

Scenario 1 modeled the 3 lists as statistically independent from each other: List 1 had a capture probability of 20%, list 2 had 25%, and list 3 had 30%.

Scenario 2 also modeled the 3 lists as statistically independent but reduced the marginal probabilities to align with the SFDPH data. List 1 sampled 14% of the population, list 2 sampled 6%, and list 3 sampled 3%.

Scenario 3 modeled conditional independence. Dependence is induced between lists 1 and 2 because of 2 additional independent binary variables, *S* (with 50% prevalence) and *A* (with 30% prevalence). The marginal probabilities for the lists are the same as in scenario 2, but the conditional probabilities change depending on whether *S* and *A* are observed. List 1 randomly samples 4.5% of the population; an additional 10% is sampled for whom *S* = 1, and an additional 15% is sampled for whom *A* = 1. List 2 samples 2% of the population; an additional 5% is sampled for whom *S* = 1, and an additional 5% is sampled for whom *A* = 1. List 3 samples 3% of the population.

Scenario 4 models conditional independence that is only partially accounted for in the TMLE approach. Three independent binary variables are simulated in addition to the 3 lists: *S* (with 50% prevalence), *A* (with 30% prevalence), and *U* (with 50% prevalence). Only *S* and *U* induce a dependence between lists 1 and 2, but only *S* and *A* are included in the **X** vector of the TMLE model to create conditional independence. List marginal probabilities are the same as in scenarios 2 and 3, but conditional probabilities differ. List 1 samples 4% of the population; an additional 10% is sampled for whom *S* = 1, and an additional 10% is sampled for whom *U* = 1. List 2 samples 4% of the population; an additional 2% is sampled for whom *S* = 1, and an additional 2% is sampled for whom *U* = 1. List 3 samples 3% of the population.

Each scenario was simulated 500 times. The margin was set at 0.02, 0.04, 0.06, 0.08, 0.1, and a data-dependent value, M.star (M.star is a dynamic value that is calculated in each simulation as the number observed on both lists 1 and 2, the 2 lists used for the TMLE, divided by the observed count from all 3 lists). Violin plots were used to visualize the distribution of size estimates for each scenario at each margin setting.

### Population size estimation

We used the R package *drpop* (19) to fit the TMLE capture-recapture model. Estimates were generated using the ward 86 and SFDPH Laboratory list pair (lists 1 and 2). The third list, the Tom Waddell clinic list, was treated as an additional list to improve the estimation of the *q*-probabilities. All measured covariates were included in the **X** vector to model conditional independence. We used SuperLearner to model covariate relationships and *q*-probabilities. The SuperLearner library included generalized additive models, generalized linear models, GLM.interaction, Ranger, and glmnet. Cross-validation was based on 2 folds. The optimal margin setting was determined from the results of the simulation analysis. These same settings were used to estimate the sizes of population subgroups.

Additional capture-recapture models were fitted for comparison. For these comparisons, we focused on models developed to correct biases from list dependence (other models have been developed to address biases resulting from violation of the other formal assumptions) (20). The R package *Rcapture* (21) was used to fit the log-linear regression models, adjusting for potential list dependencies. The R package *SparseMSE* (22) implements SparseMSE, a model designed to correct the bias resulting from small or no overlap between lists (11, 13). The R package *DGA* (23) was used to implement the decomposable graph approach. The decomposable graph approach uses Bayesian model averaging to average estimates from individual log-linear models, weighted by their posterior likelihood, into a single posterior estimate. The R package *shinyrecap* (24) was used to implement the Bayesian latent class model, which seeks to meet the identifiability constraint of list independence by conditioning on latent classes based on observed capture histories. Both the decomposable graph approach and Bayesian latent class models have shown less bias than conventional log-linear models in previous simulation studies (10, 11).

Population size estimates from all models were compared against the complete SFDPH data as the ground truth.

### Ethics

This study was reviewed and approved by the University of California, San Francisco’s institutional review board. No personally identifying information, including medical record numbers, were present in the analytical database.

## RESULTS

### Sample/study population

There were 12,507 people living with HIV in the complete SFDPH data. Of these, 2,584 were observed on the combination of the 3 lists (see Web Figure 1, available at https://doi.org/10.1093/aje/kwad231). List 1 (ward 86) accounted for 70% of the analytical sample, list 2 (SFDPH Laboratory) accounted for 28%, and list 3 (Tom Waddell clinic) accounted for 14%. Notably, there was relatively little overlap among the 3 lists. The lists differed in the distribution of covariates (Table 1). There was a greater proportion of Latino/a patients on the SFDPH Laboratory list than on either of the other lists, whereas the racial/ethnic distribution was more even on the Tom Waddell list. Females were overrepresented on all 3 lists relative to their true proportion in the surveillance data. Each list underrepresented the proportion of men who had sex with men, relative to the surveillance data, and overrepresented the proportion of people who injected drugs and the proportion of men who had sex with men and also injected drugs. Patients who injected drugs as a transmission risk category were overrepresented by the Tom Waddell list. New HIV diagnoses (in 2019) were overrepresented by the SFDPH Laboratory’s list, whereas patients who were not virally suppressed were overrepresented by the Tom Waddell list. The age distributions by list largely reflected the age distributions in the surveillance data.

**Table 1:** Characteristics of San Francisco, California, Residents Living With HIV as of December 31, 2019, by List Contributing to HIV Surveillance

			**List Contributing to HIV Surveillance**													
			**ZSFGH Ward 86**				**Tom Waddell Urban Health Clinic**				**SFDPH Laboratory**					
	**Total** **(*n* = 12,507)**		**Unique** **(*n* = 1,779)**		**With Overlap** **(*n* = 1,809)**		**Unique** **(*n* = 63)**		**With Overlap** **(*n* = 359)**		**Unique** **(*n* = 425)**		**With Overlap** **(*n* = 736)**		**Other** ^a^ **(*n* = 9,923)**	
**Characteristic**	**No.**	**%**	**No.**	**%**	**No.**	**%**	**No.**	**%**	**No.**	**%**	**No.**	**%**	**No.**	**%**	**No.**	**%**
Race/ethnicity																
White	6,569	52.5	740	41.6	754	41.7	21	33.3	113	31.5	79	18.6	178	24.2	5,627	56.7
Black	1,602	12.8	354	19.9	361	20.0	16	25.4	100	27.9	30	7.1	114	15.5	1,115	11.2
Latino/a	2,901	23.2	492	27.7	499	27.6	14	22.2	77	21.4	288	67.8	357	48.5	2,038	20.5
Other/unknown	1,435	11.5	193	10.8	195	10.8	12	19.0	69	19.2	28	6.6	87	11.8	1,143	11.5
Birth sex																
Female	743	5.9	170	9.6	171	9.5	15	23.8	58	16.2	32	7.5	75	10.2	483	4.9
Male	11,764	94.1	1,609	90.4	1,638	90.5	48	76.2	301	83.8	393	92.5	661	89.8	9,440	95.1
Age in 2019, years																
13–19	10	0.1	2	0.1	2	0.1	0	0.0	0	0.0	3	0.7	3	0.4	5	0.1
20–29	460	3.7	68	3.8	70	3.9	5	7.9	8	2.2	42	9.9	47	6.4	340	3.4
30–39	1,806	14.4	282	15.9	295	16.3	19	30.2	56	15.6	123	28.9	168	22.8	1,335	13.5
40–49	2,409	19.3	403	22.7	412	22.8	14	22.2	80	22.3	83	19.5	155	21.1	1,836	18.5
50–59	4,207	33.6	584	32.8	588	32.5	16	25.4	138	38.4	108	25.4	230	31.2	3,375	34.0
60–69	2,740	21.9	379	21.3	381	21.1	8	12.7	65	18.1	52	12.2	108	14.7	2,244	22.6
≥70	875	7.0	61	3.4	61	3.4	1	1.6	12	3.3	14	3.3	25	3.4	788	7.9
Age at diagnosis, years																
≤12	19	0.2	2	0.1	2	0.1	0	0.0	0	0.0	3	0.7	3	0.4	14	0.1
13–19	219	1.8	51	2.9	51	2.8	3	4.8	8	2.2	15	3.5	20	2.7	145	1.5
20–29	3,475	27.8	549	30.9	559	30.9	22	34.9	113	31.5	147	34.6	242	32.9	2,659	26.8
30–39	5,003	40.0	661	37.2	674	37.3	19	30.2	123	34.3	178	41.9	291	39.5	4,031	40.6
40–49	2,853	22.8	399	22.4	405	22.4	12	19.0	77	21.4	57	13.4	125	17.0	2,316	23.3
50–59	760	6.1	98	5.5	99	5.5	5	7.9	33	9.2	16	3.8	43	5.8	613	6.2
60–69	163	1.3	19	1.1	19	1.1	2	3.2	5	1.4	9	2.1	12	1.6	130	1.3
≥70	15	0.1	0	0.0	0	0.0	0	0.0	0	0.0	0	0.0	0	0.0	15	0.2
HIV risk category																
MSM	9,223	73.7	945	53.1	963	53.2	10	15.9	123	34.3	325	76.5	449	61.0	7,816	78.8
PWID	712	5.7	203	11.4	205	11.3	20	31.7	83	23.1	21	4.9	83	11.3	405	4.1
MSM-PWID	1,823	14.6	485	27.3	494	27.3	26	41.3	126	35.1	40	9.4	144	19.6	1,166	11.8
Other/unidentified	749	6.0	146	8.2	147	8.1	7	11.1	27	7.5	39	9.2	60	8.2	536	5.4
New HIV diagnosis in 2019																
No	12,275	98.1	1,752	98.5	1,772	98.0	63	95.2	355	98.9	359	84.5	659	89.5	9,798	98.7
Yes	232	1.9	27	1.5	37	2.0	3	4.8	4	1.1	66	15.5	77	10.5	125	1.3
Virally suppressed?																
No	1,300	10.4	233	13.1	240	13.3	30	47.6	75	20.9	41	9.6	90	12.2	946	9.5
Yes	10,726	85.8	1,546	86.9	1,569	86.7	33	52.4	284	79.1	379	89.2	641	87.1	8,501	85.7
Unknown	481	3.8	0	0.0	0	0.0	0	0.0	0	0.0	5	1.2	5	0.7	476	4.8

Abbreviations: HIV, human immunodeficiency virus; MSM, men who have sex with men; PWID, people who inject drugs; SFDPH, San Francisco Department of Public Health; ZSFGH, Zuckerberg San Francisco General Hospital.

^a^ "Other" encompasses all other reporting sources contributing to SFDPH HIV surveillance (not including ZSFGH Ward 86, the Tom Waddell Urban Health Clinic, and the SFDPH Laboratory).

### Simulation results

**Figure 1:**
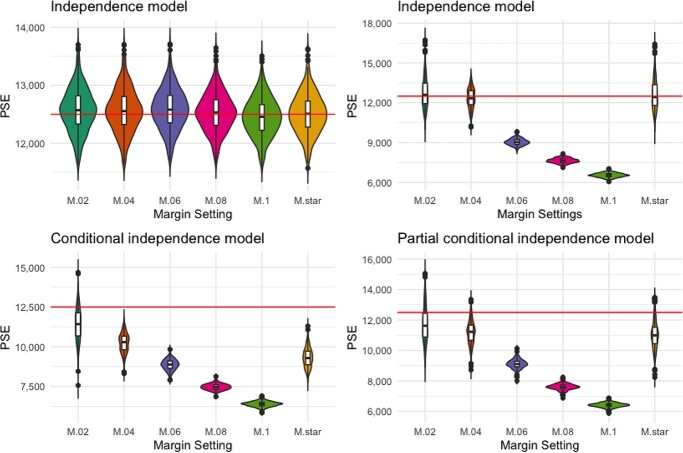
Estimated numbers of persons living with human immunodeficiency virus (HIV) as of December 31, 2019, from simulations varying the marginal probability of list overlap, San Francisco, California. A) Scenario 1; B) scenario 2; C) scenario 3; D) scenario 4. The *y*-axis refers to the estimated population size. The *x*-axis refers to the margin setting for each simulation (0.02, 0.04, 0.06, 0.08, 0.1, and M.star, a data-dependent value that is calculated as the number observed on both list 1 and list 2 divided by the observed count from all 3 lists). The red horizontal line refers to the "ground truth" estimate from the San Francisco Department of Public Health HIV surveillance office (*n* = 12,507). PSE, population size estimate.

The TMLE model was robust to different margin settings when lists were independent and sampled from the source population with at least 20% probability (Figure 1). Modeling results were sensitive to margin settings when the marginal sampling probabilities of the lists decreased. There was a clear bias-variance tradeoff with margin settings when lists were simulated to have smaller marginal capture probabilities.

In scenario 2, simulating list independence, higher margin settings (≥0.06) yielded biased distributions with a false sense of greater estimated precision. Setting the margin to 0.04 yielded minimal bias, but moderately increased the variance. The variance continued to increase when the margin was set to 0.02. The mean for M.star for this scenario was close to 0.04 (mean = 0.039; range, 0.029–0.049), but the spread of the distribution was wider than that observed when the margin was set to 0.04 because of variability in the distribution of M.star across simulations.

In scenario 3, modeling conditional independence, setting the margin to 0.02 resulted in the only distribution to cover the truth, albeit with greatest variance. The distribution was tighter when the margin was set to 0.04, but barely failed to cover the truth. Increasing margin settings yielded precise, but biased, estimates. The distribution for M.star indicated that this dynamic marginal probability fell between 0.04 and 0.06 in simulations.

In scenario 4, model misspecification yielded similar results as in previous scenarios, albeit with moderately increased variance. Setting the margin to 0.04 yielded the least biased distribution with moderate spread. Based on these results, we determined that the observed marginal probability of the overlap is distorted when covariates create a dependence between lists. For optimal performance of the TMLE model, the margin should be set to the expectation when lists are independent (0.04 for our empirical data).

### Population size estimation results

**Figure 2:**
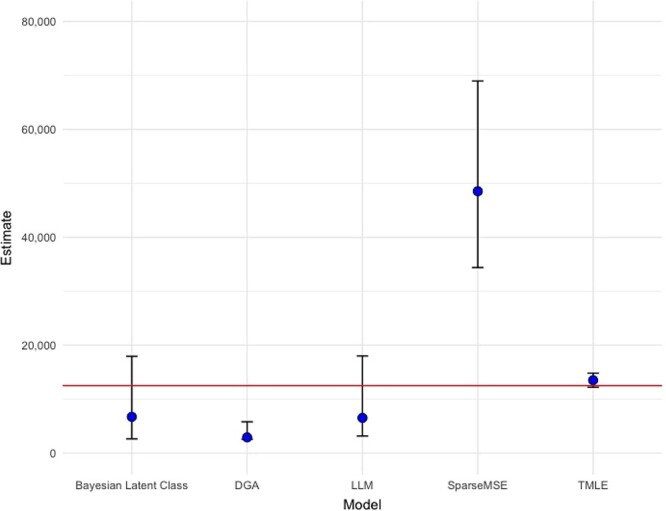
Population size estimates (points) for the number of San Francisco, California, residents living with human immunodeficiency virus as of December 31, 2019, comparing the targeted minimum loss-based estimation (TMLE) model with other common models. The horizontal red line represents the ground truth (*n* = 12,507). The log-linear model (LLM) is the best-fitting model according to the lowest Akaike information criterion value. Depending on the model, the bars represent 95% credible intervals (Bayesian latent class model, decomposable graph approach (DGA)) or 95% confidence intervals (LLM, SparseMSE, TMLE). MSE, multiple systems estimation.

Setting the margin to 0.04, the TMLE model estimated the population size to be 13,523 (95% CI: 12,222, 14,824) (Figure 2; Web Table 1). No other model produced estimates as accurate or precise. The best-fitting log-linear model included all 3 pairwise interaction terms modeling list dependence yet substantially underestimated the “true” population size (estimate = 6,536; 95% CI: 3,179, 18,010). We note, however, that the confidence interval did contain the ground truth. The Bayesian latent class model similarly underestimated the population size yet estimated a credible interval wide enough to contain the ground truth (estimate = 6,736; 95% credible interval: 2,647, 17,957). Although the intervals for both of these models contained the ground truth, the intervals were wider than the TMLE model’s interval by nearly a factor of 6. Other models either under- or overperformed, with 95% CIs excluding the ground truth.

### Population size estimates for subgroups

Subgroup estimates from the TMLE model were mixed (Table 2). The model produced accurate estimates (i.e., the 95% CI contained the ground truth) for subgroups based on new HIV diagnoses in 2019 and viral suppression. Numbers of Black patients and patients with other/unknown race/ethnicity recorded were estimated with accuracy, whereas the number of White patients was underestimated and the number of Latino/a patients was overestimated. Although male patients were accurately estimated, the number of female patients was overestimated by a factor of 1.7. Estimates based on age (age in 2019 and age at diagnosis) were either accurate or had minimal bias (the 95% CI nearly contained the ground truth). When cell sizes fell below 4% of the total count in the analytical data, the model failed to produce estimates, perhaps due to positivity violations in the context of cross-validation. The model underestimated the number of men who had sex with men and overestimated numbers in all other risk categories.

**Table 2:** Estimated Numbers of Persons in Various Population Subgroups Living With HIV as of December 31, 2019, in a Comparison of a TMLE Model With Ground Truth From the San Francisco Department of Public Health, San Francisco, California

**Characteristic**	**Observed**		**Total**		**TMLE Model**	
	**No.**	**%**	**No.**	**%**	**No.**	**95% CI**
Race/ethnicity						
White	942	36.5	6,569	52.5	3,536	2,756, 4,316
Black	487	18.8	1,602	12.8	2,139	1,555, 2,722
Latino/a	863	33.4	2,901	23.2	5,193	4,427, 5,958
Other/unknown	292	11.3	1,435	11.5	1,465	1,133, 1,796
Birth sex						
Female	260	10.1	743	5.9	1,262	1,020, 1,504
Male	2,324	89.9	11,764	94.1	11,981	10,698, 13,264
Age in 2019, years						
13–19	5	0.2	10	0.1		
20–29	120	4.6	460	3.7	635	471, 799
30–39	471	18.2	1,806	14.4	2,320	1,654, 2,986
40–49	573	22.2	2,409	19.3	2,946	2,146, 3,745
50–59	832	32.2	4,207	33.6	3,503	3,046, 3,960
60–69	496	19.2	2,740	21.9	2,141	1,805, 2,478
≥70	87	3.4	875	7.0		
Age at diagnosis, years						
≤12	5	0.2	19	0.2		
13–19	74	2.9	219	1.8		
20–29	816	31.6	3,475	27.8	4,342	3,658, 5,025
30–39	972	37.6	5,003	40.0	4,858	3,911, 5,806
40–49	537	20.8	2,853	22.8	2,226	1,721, 2,730
50–59	147	5.7	760	6.1	—^a^	—
60–69	33	1.3	163	1.3	—	—
≥70			15	0.1	—	—
HIV risk category						
MSM	1,407	54.5	9,223	73.7	5,804	4,902, 6,705
PWID	307	11.9	712	5.7	1,530	1,273, 1,787
MSM-PWID	657	25.4	1,823	14.6	2,640	2,095, 3,185
Other/unidentified	213	8.2	749	6.0	1,005	778, 1,233
New HIV diagnosis in 2019						
No	2,477	95.9	12,275	98.1	12,939	11,857, 14,022
Yes	107	4.1	232	1.9	268	107, 487
Virally suppressed?						
No	354	13.7	1,300	10.4	1,546	891, 2,202
Yes	2,225	86.1	10,726	85.8	11,845	10,635, 13,054
Unknown	5	0.2	481	3.8		

Abbreviations: CI, confidence interval; HIV, human immunodeficiency virus; MSM, men who have sex with men; PWID, people who inject drugs; TMLE, targeted minimum loss-based estimation.

^a^ Not a number (undefined value).

## DISCUSSION

Using the TMLE capture-recapture model, we estimated the number of San Francisco residents living with HIV in 2019 to be 13,523 (95% CI: 12,222, 14,824). This estimate, based on 2,584 people observed on 3 different lists, is consistent with the ground truth of 12,507 persons obtained from the SFDPH HIV surveillance office. Estimates from the TMLE model had greater accuracy and precision than those from the more commonly applied models. Most models failed to include the ground truth within their 95% CIs. The poor performance from the log-linear models was consistent with long-standing criticism that this approach cannot effectively model complex list dependencies, especially in the presence of sparce cells (10–12). More unexpected was the underperformance of the Bayesian latent class and decomposable graph approach models, both of which performed favorably in recent simulation studies (10, 11).

Our simulations indicated that the TMLE model estimates are only sensitive to margin settings when lists sample the target population with relatively low sampling probability (perhaps < 20%). When lists are independent, the margin should be set to the proportion of the sample observed on both primary lists used for estimation. When covariates induce dependence between lists, this marginal probability of being observed on both lists is also distorted, and the empirical proportion should not be used to set the margin. Instead, we recommend running a simple simulation, assuming list independence, to approximate the marginal probability. This requires a rough approximation of the target population size, which investigators and stakeholders can often provide.

Our simulation results also revealed the TMLE model’s robustness to misspecification from erroneously excluding (including) covariates that do (not) induce dependence between lists. List dependence may be induced through a simple linear combination of covariates. In this case, one might build a logistic regression model to test the association between potential covariates and overlap on the primary lists. However, covariates could induce list dependence through a more complex functional form (e.g., splines, interactions, etc.) that the investigator cannot empirically or comprehensively test a priori. Robustness to such model misspecification is therefore an attractive feature of the TMLE model.

Another attractive feature of the TMLE model is the estimation of subgroups. However, the comparison of our subgroup estimates with the ground truth warrants caution. The model reliably estimated subgroup sizes with 2 levels to the categorical variable (e.g., new HIV diagnosis, viral suppression). Other variables with more than 3 levels yielded variable results. While this function must be investigated further, investigators can assess the accuracy of estimated subgroups by summing the population sizes. In our example, the covariates with inaccurate stratified estimates were also the ones where the sum of the stratified estimates did not equal or approximate the total population size estimated from the main model.

Importantly, our data included lists with small overlap, a challenging environment for any capture-recapture method. This is further evidenced by examining the estimated empirical distribution of the estimated overlap probabilities in lists 1 and 2 (i.e., *q*_12_). A useful sensitivity analysis may be to plot size estimates across a range of margin settings.

### Limitations

Our results should be interpreted within the context of several limitations. First, as with any population size estimation study, the true population size was unknown, making it difficult to evaluate the accuracy of any estimate. The SFDPH conducts both active and passive surveillance of HIV cases. This paired with annual evaluations of the undercount in the surveillance system boosts confidence that the ground truth is a close approximation of the true number. Second, sociodemographic information recorded in clinical settings may be recorded with error, as demonstrated by a recent evaluation (25). Misclassification may be differential by race/ethnicity, age, and transmission risk category, potentially affecting the estimation of population subgroups.

### Conclusion

Estimation of the underascertainment of cases or the sizes of hidden populations is key to epidemiologic surveillance and public health programming. For example, current targets for HIV control efforts recommend that 95% of people living with HIV be tested and know their HIV status, necessitating an awareness of the size of this target population (26). Surveillance systems vary in quality, and even the most rigorous surveillance systems suffer from incompleteness. The capture-recapture method has always been appealing in leveraging information from multiple incomplete, yet overlapping, data sources. The TMLE capture-recapture model offers several advantages to move the field forward: 1) 2-sample estimation assuming conditional independence is a more reasonable assumption than 2-sample estimation assuming complete list independence with conventional log-linear modeling; 2) more than 2 lists are not required for estimation (and may be difficult to acquire), but may still be incorporated to improve estimation; and 3) drawing from semiparametric statistical theory and machine learning, bias due to model misspecification may be limited. The TMLE capture-recapture model is therefore a welcomed addition to the epidemiologist’s tool kit.

## Supplementary Material

Web_Material_kwad231

## APPENDIX 1

The steps needed to calculate the targeted minimum loss-based estimate for Ψ, the probability of being observed on any list, are as follows.

1. Obtain initial estimates of *q*_12_(*x*), *q*_1_(*x*), and *q*_2_(*x*), denoted ${\hat{q}}_{12,0}(x)$, ${\hat{q}}_{1,0}(x)$, and ${\hat{q}}_{2,0}(x)$, respectively. Set *t* = 0.
2. At step *t*, construct (so-called) clever covariates:
  $$ {H}_{12,t}=\frac{{\hat{q}}_{1,t}(X){\hat{q}}_{2,t}(X)}{{\hat{q}}_{12,t}{(X)}^2}-\frac{{\hat{q}}_{1,t}(X)}{{\hat{q}}_{12,t}(X)}-\frac{{\hat{q}}_{2,t}(X)}{{\hat{q}}_{12,t}(X)} $$
  $$ {H}_{1,t}=\frac{{\hat{q}}_{2,t}(X)}{{\hat{q}}_{12,t}(X)} $$
  $$ {H}_{2,t}=\frac{{\hat{q}}_{1,t}(X)}{{\hat{q}}_{12,t}(X)} $$

3. Regress *Y*_1_*Y*_2_ on ${H}_{12,t}$ using a no-intercept logistic model with $\mathrm{logit}\left\{{\hat{q}}_{12,t}(X)\right\}$ as an offset, obtaining the estimated coefficient ${\hat{\beta}}_{12,t}$. Set ${\hat{q}}_{12,t+1}(X)=\mathrm{expit}[\mathrm{logit}\{{\hat{q}}_{12,t}(X)\}+{\hat{\beta}}_{12}{H}_{12,t}]$.
4. Regress *Y*_1_(1 − *Y*_2_) on ${H}_{1,t}$ using a no-intercept logistic model with $\mathrm{logit}\left\{{\hat{q}}_{1,t}(X)-{\hat{q}}_{12,t+1}(X)\right\}$ as an offset, obtaining the estimated coefficient ${\hat{\beta}}_{1,t}$. Set ${\hat{q}}_{1,t+1}(X)=\min \{{\hat{q}}_{12,t+1}(X)+\mathrm{expit}[\mathrm{logit}\{{\hat{q}}_{1,t}(X)-{\hat{q}}_{12,t+1}(X)\}+{\hat{\beta}}_{1,t}{H}_{1,t}],1-{\hat{q}}_{12,t+1}(X)\}$.
5. Regress *Y*_2_(1 − *Y*_1_) on ${H}_{2,t}$ using a no-intercept logistic model with $\mathrm{logit}\left\{{\hat{q}}_{2,t}(X)-{\hat{q}}_{12,t+1}(X)\right\}$ as an offset, obtaining the estimated coefficient ${\hat{\beta}}_{2,t}$. Set ${\hat{q}}_{2,t+1}(X)=\min \{{\hat{q}}_{12,t+1}(X)+\mathrm{expit}[\mathrm{logit}\{{\hat{q}}_{2,t}(X)-{\hat{q}}_{12,t+1}(X)\}+{\hat{\beta}}_{2,t}{H}_{2,t}],1+{\hat{q}}_{12,t+1}(X)-{\hat{q}}_{1,t+1}(X)\}$.
6. Update *t → t* + 1. Repeat steps 2–6 until convergence (e.g., until ${\max}_j\left|{\hat{\beta}}_{j,t+1}\right|\le \mathrm{\varepsilon}$).
7. Finally, set ${\hat{\Psi}}_{\mathrm{tmle}}={\left[{Q}_N\left\{\frac{{\hat{q}}_1^{\ast }(X){\hat{q}}_2^{\ast }(X)}{{\hat{q}}_{12}^{\ast }(X)}\right\}\right]}^{-1}$, where ${\hat{q}}_j^{\ast }$ are estimates obtained after convergence.

In the above notation, *q*_12_(*x*), *q*_1_(*x*), and *q*_2_(*x*) are the probabilities of selection on lists 1 and 2, list 1, and list 2, respectively; *Y*_1_ is the binary outcome variable for appearing on list 1, *Y*_2_ is the binary outcome variable for appearing on list 2, and *Y*_1_*Y*_2_ is the binary outcome variable for appearing on both list 1 and list 2; ${\hat{\Psi}}_{\mathrm{tmle}}$ is the targeted minimum loss-based estimate for the target parameter, the probability of selection on at least 1 list; and ${Q}_N$ is the empirical measure of the observed data distribution $Q$.
