# Supplementary material for: Evaluating a Targeted Minimum Loss-Based Estimator for Capture-Recapture Analysis: An Application to HIV Surveillance in San Francisco, California
Source: Am J Epidemiol. 2023 Nov 17;193(4):673–83. doi: 10.1093/aje/kwad231 (PMC10999650; doi:10.1093/aje/kwad231)
Supplement: Web_Material_kwad231 [file web_material_kwad231.pdf]

WEB MATERIAL

Evaluating a Targeted Minimum Loss-Based Estimator for Capture-Recapture Analysis: An Application to HIV Surveillance in San Francisco, California

Paul Wesson, Manjari Das, Mia Chen, Ling Hsu, Willi McFarland, Edward Kennedy, and Nicholas P. Jewell

Table of Contents

Web Figure 1… Page 2

Web Table 1… Page 3

Web Figure 1. Venn diagram of list information used for capture-recapture analysis of the number of San Francisco residents living with HIV as of December 31, 2019.

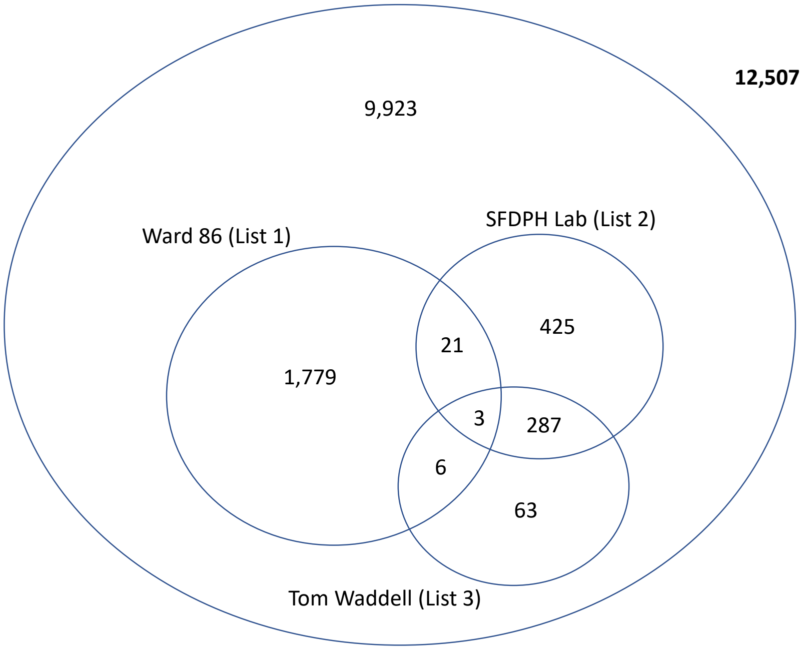

HIV, human immunodeficiency virus; SFDPH, San Francisco Department of Public Health.

Web Table 1. Population size estimation results for the number of San Francisco residents living with HIV as of December 31, 2019

Model	Estimate	95% CI	AIC
Truth	*12,507*	--	--
Observed	*2,584*	--	--
TMLE	13,523	12,222 – 14,824	NA
Independence	6,796	6,238 – 7,434	1,462.576
L1*L2, L3	5,109	4,736 – 5,537	1,262.176
L1*L3, L2	3,057	2,931 – 3,205	817.593
L2*L3, L1	48,542	34,975 – 70,350	64.974
L1*L3, L2*L3	21,264	11,252 – 50,433	63.019
L1*L2, L2*L3	38,588	26,335 – 60,190	64.188
L1*L2, L1*L3	2,677	2,647 – 2,712	69.238
L1*L2, L1*L3, L2*L3	6,536	3,179 – 18,010	56.233
DGA	2,929	2,584 – 5,816	NA
Bayesian Latent Class	6,736	2,647 – 17,957	NA
SparseMSE	48,542	34,403 – 68,962	64.97

Abbreviations: AIC, Akaike information criterion; CI, confidence interval; DGA, decomposable graph approach; HIV, human immunodeficiency virus; L, list; MSE, multiple systems estimation; NA, not applicable; TMLE, targeted minimum loss-based estimation.
